# Control of Toxin-Antitoxin Systems by Proteases in *Mycobacterium Tuberculosis*

**DOI:** 10.3389/fmolb.2021.691399

**Published:** 2021-05-17

**Authors:** Patricia Bordes, Pierre Genevaux

**Affiliations:** Laboratoire de Microbiologie et de Génétique Moléculaires, Centre de Biologie Intégrative, Université de Toulouse, CNRS, UPS, Toulouse, France

**Keywords:** AAA+ proteases, proteasome, toxin-antitoxin system, mycobacterium, protein degradation

## Abstract

Toxin-antitoxin (TA) systems are small genetic elements composed of a noxious toxin and a counteracting cognate antitoxin. Although they are widespread in bacterial chromosomes and in mobile genetic elements, their cellular functions and activation mechanisms remain largely unknown. It has been proposed that toxin activation or expression of the TA operon could rely on the degradation of generally less stable antitoxins by cellular proteases. The resulting active toxin would then target essential cellular processes and inhibit bacterial growth. Although interplay between proteases and TA systems has been observed, evidences for such activation cycle are very limited. Herein, we present an overview of the current knowledge on TA recognition by proteases with a main focus on the major human pathogen *Mycobacterium tuberculosis*, which harbours multiple TA systems (over 80), the essential AAA + stress proteases, ClpC1P1P2 and ClpXP1P2, and the Pup-proteasome system.

## General Overview of Toxin Antitoxin Systems in *M. tuberculosis*

The bacterium *Mycobacterium tuberculosis*, the causative agent of tuberculosis, is a major public health problem accounting for over 1.5 million deaths per year. The emergence of multidrug resistant (MDR) and extensively drug-resistant (XDR) *Mtb* strains has significantly challenged current tuberculosis treatments and increase the need for new treatment strategies (WHO report 2018; www.who.int/tb/data). The ability to sense and tolerate multiple host derived stresses, evade host defenses and persist within infected hosts is central to the pathogenicity of *M. tuberculosis*. Therefore, deciphering molecular mechanisms underlying stress tolerance and sensing in *M. tuberculosis* is critical for developing new strategies to fight tuberculosis.

*M. tuberculosis* strains possess a remarkably high number of toxin-antitoxin (TA) systems in their genome (Ramage et al., 2009; Akarsu et al., 2019; Tandon et al., 2019). As an example, the most studied laboratory strain H37Rv encodes for more than 80 TA systems and it has been proposed that such systems could contribute to it pathogenesis (Sala et al., 2014). Classical TA systems are small genetic modules composed of a deleterious toxin and an antitoxin that neutralizes the effects of the toxin. TA systems are organized into operons and are widely distributed throughout the bacterial genome (Van Melderen, 2010). Toxins generally target essential functions of the host bacterium, such as translation, replication, membrane integrity or peptidoglycan synthesis, causing growth to slow down and eventually leading to cell death (Page and Peti, 2016; Harms et al., 2018; Wilmaerts et al., 2018). TA systems are often found on plasmids, for which they were designated as addiction modules since they are involved in their stabilization by inhibiting growth of daughter cells that would not have inherited the plasmid carrying the TA system (Ogura and Hiraga, 1983). The roles of chromosomal TA systems remain largely unknown. However, they have been associated with protection against phage infection or stabilization of genomic regions (Pecota and Wood, 1996; Fraikin et al., 2020; Peltier et al., 2020). In addition, they also contribute to the virulence and persistence of pathogenic bacteria *in vivo* in infection models (Helaine et al., 2014; Lobato-Márquez et al., 2016; Agarwal et al., 2020).

There are seven known classes of TA systems depending on the nature of the antitoxin and its mode of action on the toxin, with the toxin always being a protein. In Type I systems, the antitoxin is a small anti-sense RNA that forms a duplex with the toxin's mRNA to inhibit toxin production (Brantl, 2012). Type III antitoxins are RNAs that inactivate the toxin by forming a complex (Blower et al., 2012). For type IV, the antitoxin suppresses the toxicity of the toxin by stabilizing its targets (Masuda et al., 2012), and Type V is represented by the GhoT-GhoS system, in which the antitoxin inhibits the toxin by specific cleavage of its mRNA (Wang et al., 2012). In the type VI SocAB system of *Caulobacter crescentus* (Aakre et al., 2013)*,* the SocB toxin is responsible for the essentiality of the *clpX* and *clpP* genes in this bacterium, and the SocA antitoxin serves as an adaptor protein to address the SocB toxin to the ClpXP AAA^+^ protease. For the recently identified type VII, the antitoxin neutralizes the toxin through post-translational modification of the toxin such as phosphorylation or oligoAMPylation (Songailiene et al., 2020; Yu et al., 2020). The most characterized TA systems are type II systems (Xie et al., 2018). In this case, the antitoxin is a protein that interacts with the toxin to form a complex in which the toxin is inactive (Van Melderen, 2010). They generally are auto-repressor of their own transcription, most often in complex with the toxin (Fraikin et al., 2020).

TA systems present in *M. tuberculosis* genome are mostly type II TA systems, including at least 51 VapBC systems, 10 MazEF, 1 PemIK, 2 RelBE, 1 YefM/YoeB, 3 HigBA, and 2 ParDE family members, as well as several newly identified systems including PezAT, PhoAT-PhoH2 and MbcTA. Besides, the DarTG system is a hybrid typeII/IV system and MenTA3 a type VII (Cai et al., 2020; Yu et al., 2020). *M. tuberculosis* TA systems are generally located within regions of horizontal gene transfer together with genes involved in virulence, dormancy, regulation or cell signaling (Ramage et al., 2009; Sala et al., 2014; Wang et al., 2015), suggesting that they could also contribute to the success of *M. tuberculosis* as a human pathogen. A substantial number of *M. tuberculosis* toxins have been cloned and showed toxicity when expressed in *E. coli* or in mycobacteria (Ramage et al., 2009; Sala et al., 2014; Agarwal et al., 2018; Akarsu et al., 2019). Besides, transcription of several *M. tuberculosis* TA systems were shown to be induced under various stress conditions including drug exposure, hypoxia, heat-shock, DNA damages (Sala et al., 2014; Tiwari et al., 2015; Gupta et al., 2017; Agarwal et al., 2018), and gene deletion mutants *ΔvapC22*, *ΔvapBC3/4/11* and *ΔmazF3/6/9* are strongly impaired in host infection (Tiwari et al., 2015; Agarwal et al., 2018; Deep et al., 2018; Agarwal et al., 2020). Even though transcriptional induction of TA systems does not necessarily reflect toxin activation (LeRoux et al., 2020), these data suggest that toxins could modulate bacterial growth depending on environmental conditions, and thus contribute to *M. tuberculosis* physiology and virulence (Sala et al., 2014). This also implies that their toxic activity must be tightly regulated in order not to be detrimental for bacterial survival. Since all the TA systems described so far in *M. tuberculosis* encode protein toxins and antitoxins, one of the main control mechanism that could enable a fast change of Toxin/Antitoxin ratios in response to changing cellular conditions is differential proteolysis (Jenal and Hengge-Aronis, 2003; Molière and Turgay, 2013).

## Proteolytic Regulation of Toxin Antitoxin Systems

In bacteria, protein turnover is mainly achieved by multi-subunit machines known as AAA + proteases and the proteasome. It has been proposed that under certain conditions, type II antitoxins are degraded by AAA^+^ proteases Lon or Clp, which could result in lifting the repression of the operon and activation of the toxin (Van Melderen et al., 1994; Jensen and Gerdes, 1995; Koga et al., 2011). Except for the recently described degradation of a ParE-like antitoxin of *Microcystis aeruginosa* PCC 7806 by a caspase homolog protease (Klemenčič et al., 2021), only AAA^+^ proteases (ClpAP, ClpCP, ClpXP, Lon) have been involved in antitoxin degradation in bacteria (Van Melderen et al., 1994; Lehnherr and Yarmolinsky, 1995; Aizenman et al., 1996; Prysak et al., 2009; Donegan et al., 2010; Diago-Navarro et al., 2013; Muthuramalingam et al., 2016; Dubiel et al., 2018; Zhou et al., 2021).

How antitoxins are targeted to degradation remains largely unknown. Some appeared to be more susceptible to proteases due to their hydrophobic or flexible C-termini or to the presence of intrinsically disordered central regions (Yamaguchi et al., 2011). In some cases, antitoxin degradation might be assisted by specific adaptors, as it is the case for the *Staphylococcus aureus* adaptor protein TrfA that assists ClpCP-mediated degradation of the MazE antitoxin (Donegan et al., 2014), or even modulated by DNA (Dubiel et al., 2018; LeRoux et al., 2020). Although antitoxins are generally more sensitive to proteolysis than their cognate toxins, it is not known whether an antitoxin within a preformed TA complex can be directly targeted by proteases to induce toxin activation *in vivo*. Although it was suggested *in vitro* that an excess of the Lon protease could disrupt a preformed DinJ-YafQ complex *in vitro* (Ruangprasert et al., 2017), there is significant evidence showing that once a stable TA complex is formed, the antitoxin is generally protected from degradation (Dubiel et al., 2018; LeRoux et al., 2020; Lunge et al., 2020). Other attractive possibilities would be that certain stress conditions or alternative factors such as adaptors or chaperones would trigger TA complex dissociation in order to proteases to get access to their substrate antitoxin. In addition, cross-talks between multiple endogenous antitoxins from the same family (as found in *M. tuberculosis*) could also be involved in TA complex unstability. Indeed, non-cognate interactions between TA systems could lead to the formation of less stable non-cognate complexes with increased sensitivity to proteases, and potentially affect the promoter binding activities of TA complexes. Intriguingly, Leroux and colleagues (2020) recently showed for several chromosomal TA systems of *E. coli* that antitoxin degradation by different stresses led to the transcriptional de-repression of their TA operon but in contrast, did not induce in any detectable toxin activation, thus further raising questions about how toxins can be activated and what is the role played by proteases in this process.

## Mycobacterial AAA^+^ Proteases

In *M. tuberculosis*, two cytosolic AAA + proteases have been identified: ClpC1P1P2 and ClpXP1P2. AAA + proteases combine a central ring-shaped peptidase ClpP, together with a regulatory hexameric ring-shaped unfoldase (ClpX or ClpC1) to bind and translocate the substrate to the central pore of the peptidase (Sauer and Baker, 2011). *M. tuberculosis* is one of the few bacteria that possess two essential *clpP* genes, which encode a hetero-oligomeric peptidase from a pair of homo-heptameric rings ClpP1P2 (Leodolter et al., 2015; Alhuwaider and Dougan, 2017; Vahidi et al., 2020). Interestingly, ClpX and ClpC1 unfoldases only interact with the ClpP2 ring surface (Leodolter et al., 2015). *M. tuberculosis* also encodes the membrane-bound AAA + protease FtsH that harbours peptidase and unfoldase activities on one single polypeptide. Apart from the fact that it can functionally complement some activities of *E. coli* FtsH (Srinivasan et al., 2006), its function in *M. tuberculosis* is poorly understood and transposon saturated mutagenesis did not firmly established its essentiality (Sassetti et al., 2003; DeJesus et al., 2017).

The Clp proteases of *M. tuberculosis* have been shown to be induced by stress conditions such as starvation or streptomycin exposure (Gupta et al., 2017). Moreover, *clpC1* and *clpP1P2* expression is directly activated under stress conditions by the regulator ClgR, a stress regulator essential during macrophage infection and the reaeration response (Estorninho et al., 2010; Sherrid et al., 2010). Noticeably, ClgR is itself a substrate for ClpP1P2 proteolytic activity, indicating that *clp* genes regulation is tightly controlled in *M. tuberculosis* (Sherrid et al., 2010; Yamada and Dick, 2017). Both AAA + unfoldases ClpX and ClpC1 are essential for the growth of *M. tuberculosis* H37Rv (DeJesus et al., 2017; Lunge et al., 2020; Kester et al., 2021). ClpX has been shown to be involved in DNA replication and in cell division in *M. tuberculosis* (Dziedzic et al., 2010; Kester et al., 2021), and a global protein expression profiling following *clpC1* gene silencing in *M. tuberculosis* showed that ClpC1P1P2 acts on several essential proteins involved in central metabolism and cell wall biosynthesis (Lunge et al., 2020). Similar to the ClpC1 homologue ClpA in *E. coli*, a small subset of ClpC1-sensitive proteins harbour typical N-end degrons composed of four residues (Tyr, Phe, Trp, and Leu) known to be recognized by the ClpS adaptor in *E. coli* (Erbse et al., 2006). However, the vast majority of ClpC1P1P2-regulated proteins in *M. tuberculosis* have disorder-promoting residues (Pro, Arg, Gly, Gln, Ser, Glu, Lys, and Ala) within their terminal 15-aa region, and it was demonstrated that this is a critical feature for ClpC1P1P2 degradation of the small heat shock protein Hsp20 in *M. tuberculosis* (Lunge et al., 2020). ClpC1 recognition can also rely on the phosphorylation of an internal residue as shown for the anti-sigma factor RseA (Barik et al., 2010).

Depletion or drug-dependent inhibition of ClpP1P2 in *M. tuberculosis* identified four protein clients with putative degrons, namely ClgR, tmRNA SsrA and the two regulators WhiB1 and CarD, all four degradation signals located at the C-terminus and enriched in hydrophobic residues (Raju et al., 2012; Yamada and Dick, 2017). Comparison of their C-terminal with known *E. coli* ClpX subtrates (Flynn et al., 2003) suggests that these substrates (with the exception of WhiB1) might be recognized by ClpX (Alhuwaider and Dougan, 2017). Other studies revealed that the membrane-associated anti-σ factor RsdA of *M. tuberculosis* was a ClpXP1P2 substrate and its Val-Ala-Ala internal degron was similar to the SsrA-tag (Jaiswal et al., 2013). More recently, similar degron sequences were identified in the cytoplasmic sequence of three other anti-σ factors of *M. tuberculosis* but instead of leading to proteolysis, they affect the unfoldase activity of ClpX to regulate the inactive σ/anti-σ complex and thus modulate gene expression (Joshi et al., 2019). This is reminiscent of ClpX interaction with FtsZ that does not lead to altered intracellular levels of FtsZ but rather to an inhibition of Z-ring assembly in *M. tuberculosis* (Dziedzic et al., 2010).

Interestingly, ClpC1 is the target of antimycobacterial peptides such as cyclomarin A or lassomycin, and has emerged as an promising drug target (Lupoli et al., 2018; Fraga et al., 2019; Maurer et al., 2019). More generally, the activation, repression of modification of ClpP mechanism of action has been the focus of many studies to identify new antibiotics (Ye et al., 2016; Moreno-Cinos et al., 2019). For instance, acyldepsipeptides (ADEPs) kill *M. tuberculosis* by preventing the binding of AAA + regulatory unfoldases to ClpP1P2 (Famulla et al., 2016), peptide boronates prevent growth of *M. tuberculosis* by inhibition of ClpP1P2 active sites (Akopian et al., 2015) and pyrazinamide prodrug triggers ClpC1P1P2 dependent degradation of the essential PanD protein by modifying its oligomeric state (Gopal et al., 2020). To date, no adaptor protein has been described for *M. tuberculosis* ClpX (Alhuwaider and Dougan, 2017), although the essential DNA maintenance protein Single-Stranded DNA Binding protein (SSB) is able to activate ClpXP1P2 proteolytic activities (Kester et al., 2021). The only adaptor described so far in mycobacteria, ClpS, inhibits ClpC1-dependent unfolding and degradation of substrate SsrA, but also enhances the degradation of an N-end rule model substrate *in vitro* (Marsee et al., 2018; Ziemski et al., 2020).

## Interplay Between AAA^+^ Proteases and Toxin Antitoxin Systems

**FIGURE 1:**
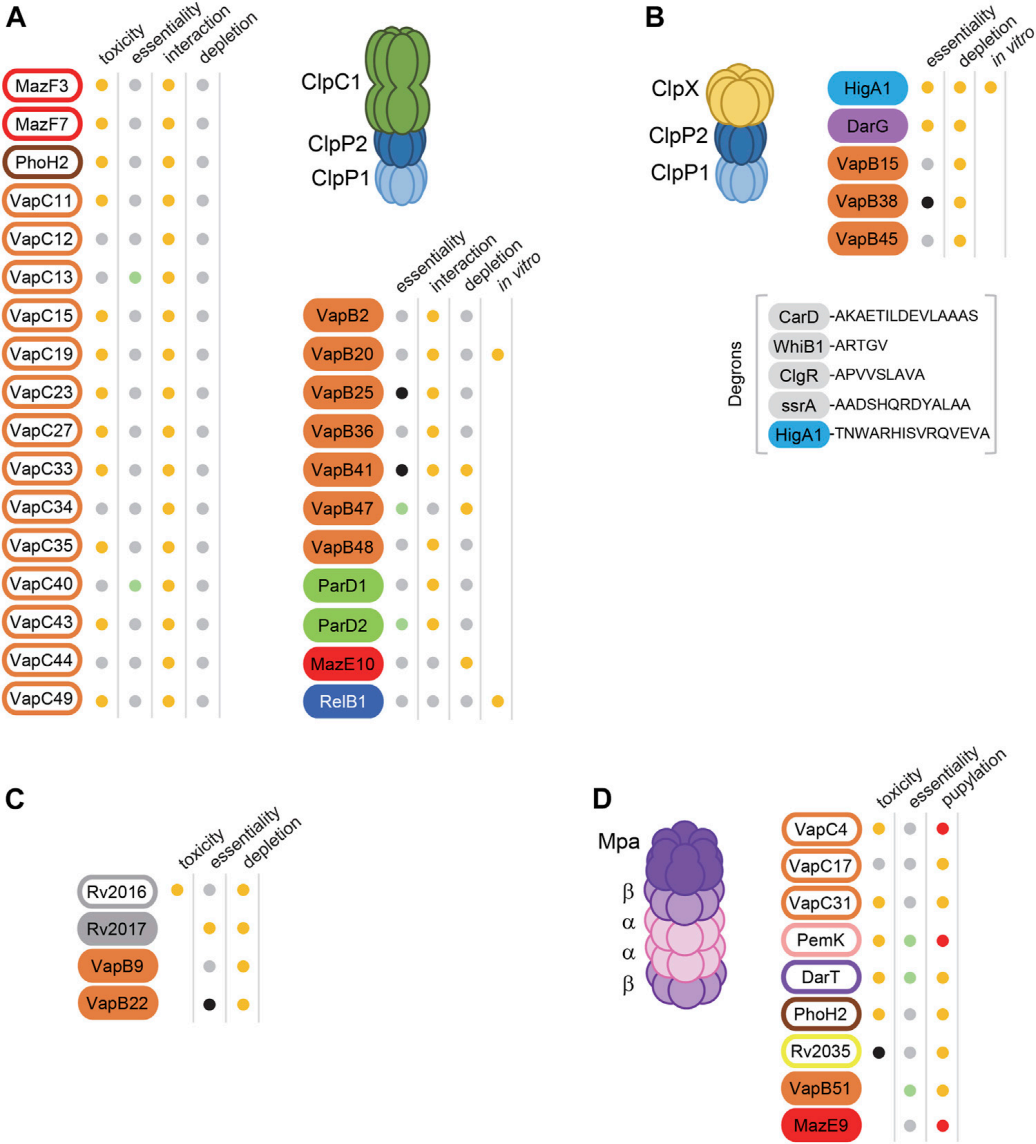
Proteolytic regulation and recognition of Toxin-Antitoxin systems in *M. tuberculosis*. TA families are indicated by different colors as followed: Orange for VapBC, Green for ParDE, pink for PemIK, red for MazEF, dark blue for RelBE, purple for DarTG, bright blue for HigBA, brown for PhoAT-H2, yellow for ArsR-COG3832 and grey for unknown. Toxins and Antitoxins are indicated by filled and open rounded rectangles, respectively. Toxin and Antitoxin proteins are candidate substrates for proteases, **(A)** ClpC1P1P2 **(B)** ClpXP1P2, **(C)** ClpP1P2 (the associated chaperone subunit, either ClpX or ClpC1 is to be determined), **(D)** the Mpa-proteasome. Known degrons for ClpXP1P2 are indicated in **(B)** under brackets. Functional properties were indicated in the columns adjacent to the toxins and antitoxins, *i.e*., toxicity, essentiality (essential), interaction, accumulation following protease depletion (depletion), *in vitro* degradation (*in vitro*) or pupylation. Toxicity: orange dots mean toxic when overexpressed (Ramage et al., 2009; Sala et al., 2014; Agarwal et al., 2018; Akarsu et al., 2019) in at least one bacterial host (*M. tuberculosis*, *M. smegmatis* or *E. coli*), grey dots nontoxic and black dot non tested. Essentiality (DeJesus et al., 2017): orange dots mean essential, green dots mean growth advantage when mutated, grey dots non-essential, black dots mean uncertain or non-tested. Interaction with chaperone subunit ClpC1 *in vivo* (Ziemski et al., 2020): orange dots mean interaction and grey dots no interaction. Depletion of *clpP1P2*, *clpP2* or *clpC1* (Raju et al., 2014; Lunge et al., 2020): orange dots mean protein stabilization and grey dots mean no detectable protein changes. *In vitro* degradation assays (Ziemski et al., 2020; Texier et al., 2021): orange dots mean degradation. Pupylation: orange dots mean pupylated under routine culture conditions (Festa et al., 2010), red dots mean pupylated by reconstituted system in *E. coli* and/or *in vitro* (Chi et al., 2018).

Several *M. tuberculosis* antitoxins have been demonstrated as ClpXP1P2 or ClpC1P1P2 substrates. One of the first proteomic study of ClpP1P2-dependent protein substrates in *M. tuberculosis* following depletion of endogenous ClpP1P2 identified 6 antitoxins as putative ClpP1P2 substrates, namely MazE10, VapB22, VapB9, VapB41, Rv2017 and HigA1 (Raju et al., 2014); Figure 1). Among these antitoxins, MazE10 was later identified as a likely ClpC1P1P2 substrate *in vivo*, together with VapB47 (Lunge et al., 2020); Figure 1). Note that both MazE10 and VapB47 antitoxins have disorder-promoting residues at their C-terminal end, which was suggested to be important for ClpC1 recognition (Lunge et al., 2020). It is striking that when we applied similar search for disordered C-terminal ends, we found that more than 60% of the antitoxins of *M. tuberculosis* possess this type of C-terminal region, thus suggesting that disordered C-terminal ends could indeed contribute to recognition by proteases. Note that a table presenting the C-terminal ends of all known *M. tuberculosis* antitoxins can be found in Texier and colleagues (Texier et al., 2021). The HigA1 antitoxin possesses a typical C-terminal ClpX degron with two crucial hydrophobic last residues Val-Ala that were recently shown to be the recognition sequence of HigA1 by ClpX in *M. tuberculosis* (Texier et al., 2021). Noticeably, the Rv2017 antitoxin contains a ClpX-like degron with two hydrophobic residues located at its extreme C-terminal part (Ala-Ile), suggesting that it could also be recognized by ClpX. The last four ClpP1P2-dependent antitoxins, namely VapB9, VapB22, VapB41 harbor different C-terminal ends and no other common feature could be detected (Raju et al., 2014). This suggests that the ClpXP1P2 and ClpC1P1P2-dependent degradation signals are not restricted to typical degrons, and that their degradation might rely on post-translational modifications or on unknown adaptors (Trentini et al., 2016).

In a recent CRISPRi study performed in *M. tuberculosis*, 4 antitoxins, namely VapB15, VapB38, VapB45 and DarG, were found to be up-regulated upon ClpP2 depletion but not upon ClpC1 depletion in *M. tuberculosis* (Lunge et al., 2020), suggesting that these antitoxins might be ClpXP1P2 substrates. This is particularly likely for VapB45, which contains a typical ClpX-degron at its C-terminus (Ala-Ala). A systematic search for ClpC1 interactors based on the bacterial adenylate cyclase two-hybrid (BACTH) screen in *E. coli* showed that type II TA systems of *M. tuberculosis* are one of the largest group of ClpC1 interacting partners (Ziemski et al., 2020). Members of the VapBC, MazEF and ParDE TA families were identified, with VapBC systems being the most abundant pairs found to be interacting partners of the ClpC1P1P2 protease complex (20 out of the 51 known VapBC pairs; Figure 1). Both VapB20 and the RelB1 antitoxins were further confirmed to be specific substrates for ClpC1P1P2 and not ClpXP1P2 using *in vitro* degradation assays (Ziemski et al., 2020), thus suggesting that ClpC1 and ClpX chaperones may not share substrate recognition motifs. Note that VapC20 or RelE1 toxin form stable complexes with their cognate antitoxin in which the antitoxin is protected from degradation, further raising questions about how the toxin could be freed from the antitoxin in order to be activated (see above comments).

Interestingly, the HigA1 degron has been the only ClpX-dependent recognition sequence identified so far for a mycobacterial antitoxin (Texier et al., 2021). HigA1 is part of the tripartite toxin-antitoxin-chaperone (TAC) system of *M. tuberculosis* that includes a cognate SecB-like chaperone (SecB^TA^). In most Gram-negative bacteria, SecB targets presecretory proteins to the Sec translocon located at the inner membrane (Bechtluft et al., 2010). In contrast with classical two-component TA systems, the TAC toxin-antitoxin pair is tightly controlled by SecB^TA^, through a direct interaction between the chaperone and an unusual aggregation-prone C-terminal extension of the antitoxin HigA1, named ChAD (chaperone-addiction) (Bordes et al., 2011; Bordes et al., 2016; Guillet et al., 2019). Binding of SecB^TA^ to the ChAD of the antitoxin protects HigA1 from aggregation and degradation. Remarkably, both SecB^TA^ binding site and ClpX degron are located within the same ChAD region of the antitoxin (with different residues being involved). These data suggest that under certain stress conditions, SecB^TA^ could be hijacked by protein substrates (either aggregated pre-proteins or specific exported proteins) and the HigA1 antitoxin could be degraded by ClpXP1P2, which could lead to a transient activation of the HigB1 toxin until normal growth conditions resume.

The analysis of the amino acid sequence of *M. tuberculosis* antitoxins suggests that only VapB19, VapB41, VapB44, VapB45, Rv1990c, Rv2017 and HigA2 antitoxins possess a putative HigA1-like degron sequence with at least two hydrophobic residues at their extreme C-terminus (respectively, Leu-Ala, Ala-Ala-Leu, Ala-Val, Ala-Ile-Ala-Ala, Val-Phe-Val, Ala-Ile and Leu-Ala). These hydrophobic residues are mainly non-polar aliphatic (Ala, Val, Leu, Ile) as usually observed in C-terminal ClpX degrons, except for Rv1990c that presents an aromatic phenylalanine. In addition, VapB41, VapB44, VapB45 and Rv1990c also possess an acidic residue before the hydrophobic end, as found in HigA1. This suggests that these antitoxins could also be recognized by *M. tuberculosis* ClpX. In addition, five poorly conserved VapB antitoxins (VapB19, VapB23, VapB28, VapB30 and VapB34) share a highly similar extreme C-terminus, with hydrophobic residues following an arginine (Arg-Gly-Leu-Pro-Ala-Pro, Arg-Gly-Leu-Pro-Ala or Arg-Leu-Gly-Leu-Ala motifs), suggesting that these antitoxins could share similar degrons (Texier et al., 2021).

Remarkably, toxins were also identified as proteases targets or putative substrates. Indeed, while only 6 VapB antitoxins were identified as ClpC1 interactors *in vivo*, the remaining 14 interactors were VapC toxins (Lunge et al., 2020). These intriguing results suggest that toxins might themselves be the targets of proteases or in contrary, act as *bona fide* protease adaptors for their cognate antitoxins and thus being actor of their own activation. Moreover, the degradation of the toxin could also be part of a bacterial strategy to resume growth after TA system activation, as demonstrated for the type I toxin HokB in *E. coli* (Wilmaerts et al., 2019). In this case, awakening of HokB-induced persister cells was shown to require the degradation of HokB monomers by the periplasmic stress protease DegQ. Whether such mechanism exists in *M. tuberculosis* remains to be determined.

## Possible Link Between Toxin Antitoxin and the Pup-Proteasome System

Another peculiarity of Actinomycetes is to possess a eukaryotic-like proteasome (Festa et al., 2010; Müller and Weber-Ban, 2019). The mycobacterial proteasome consists of a highly conserved central peptidase core particle (20S CP) composed of 28 subunits (2 heptameric inner rings composed of PcrB subunits, and 2 heptameric outer rings composed of PcrA subunits), which is gated and interact with ring-shaped activators to form a fully active protease capable of degrading specific sets of cellular substrates (Figure 1). With the mycobacterial proteasomal AAA + Mpa, the proteasome targets substrates that have been post-translationally modified with Pup (prokaryotic ubiquitin-like protein) by a dedicated ligase PafA (Pearce et al., 2008; Burns et al., 2009). Hundreds of *M. tuberculosis* pupylated proteins, which include Mpa and PafA, have been identified by proteomics studies, even though many of them are not degraded under normal growth conditions (Festa et al., 2010; Müller and Weber-Ban, 2019). This could be reminiscent of the Pup degradation-independent regulatory role in several bacterial species (Elharar et al., 2014; Küberl et al., 2016).

In mycobacteria, Pup goes through a deamidation step by the Dop enzyme before it can be attached to a target by PafA, and Pup can also be removed from tagged substrates by Dop, as well or transferred between substrates by PafA (Burns et al., 2010; Imkamp et al., 2010; Zhang et al., 2017). These enzymatic activities must be tightly regulated in order to avoid useless cycles of pupylation/depupylation and even though little is known about these regulations, it was shown that Pup-free Dop is depleted under stress conditions leading to accelerated proteasomal degradation (Elharar et al., 2016) and that the AAA + protease ClpC1P1P2 is responsible for the depletion of Pup-free Dop under starvation conditions (Hecht et al., 2020). Dop, PafA, Pup, Mpa, and 20S CPs constitute the core “Pup-proteasome system” (PPS). Two other partners of the 20S CP have been described in *M. tuberculosis*: The non-ATPase activator Bpa (also known as PafE) could address unstructured substrates to proteasomal degradation (Delley et al., 2014; Jastrab et al., 2015), and the AAA + Cpa (a Cdc48-like protein), that interacts with the 20S core but for which no degradation substrate has been identified yet (Ziemski et al., 2018). PPS mutants of *M. tuberculosis* are viable but are highly attenuated in a mouse infection model (Darwin et al., 2003; Gandotra et al., 2007), are highly sensitive to NO due to the failure to degrade a single pupylated substrate, Log (MacMicking et al., 1997; Samanovic et al., 2015) and are unable to use nitrate as a nitrogen source (Becker et al., 2019). Moreover, a *bpa* mutant displays a slow growth *in vitro* and in mice and is hypersensitive to heat shock (Jastrab et al., 2015). Anti-TB drugs targeting the mycobacterial proteasome are promising but they face the challenge of being highly selective in order not to inhibit the human proteasome (Lin et al., 2013; Bibo-Verdugo et al., 2017; Zhan et al., 2019).

Although AAA^+^ proteases are the main proteases shown to be involved in antitoxin degradation (Muthuramalingam et al., 2016), several studies suggest that the PPS could also be involved in the regulation of TA systems. Indeed five toxins, namely Rv2035, DarT, PhoH2, VapC17 and VapC31, and the VapB51 antitoxin are part of the *M. tuberculosis* pupylome under standard laboratory growth conditions culture conditions (Festa et al., 2010). Note that it remains to be determined whether these proteins are directly pupylated and if pupylation leads to their degradation by the PPS. In addition, the reconstitution of a pupylation system in *E. coli* and *in vitro* showed that the VapC4 and PemK toxins, and the MazE9 antitoxin could also be pupylated (Chi et al., 2018). Intriguingly, PhoH2 is the only toxin potentially regulated both by the proteasome and ClpC1P1P2 (Figure 1), possibly to ensure low toxin level. The fact that there are more potentially pupylated toxins than antitoxins is striking and suggests that some toxins might be differently regulated by proteasomal degradation and antitoxin inhibition (Burns et al., 2010). Yet, the *M. tuberculosis* pupylome was performed under standard laboratory growth conditions and we cannot exclude that more antitoxins could be pupylated under certain stresses, as growth conditions were shown to modify the abundance of pupylated proteins (Becker et al., 2019). The fact that Bpa could drive proteasomal degradation of partially or totally unfolded proteins, which is a property of many antitoxins, suggests that other proteasome activators could be involved in TA proteins turnover (Jastrab et al., 2015). Finally, there could also be a link between the master regulator PafBC, which is encoded within the PPS gene locus and the regulation of TA systems, as the VapB antitoxin of *M. smegmatis* was shown to be part of the pafBC regulon (Müller et al., 2018).

## Concluding Remarks

A substantial number of *M. tuberculosis* antitoxins are *bona fide* substrates of AAA^+^ proteases, both *in vivo* and *in vitro* (Figure 1). Yet, there is very little knowledge about recognition signals within antitoxins and degron sequences are just beginning to emerge. In addition, it is not known whether proteases directly play a role in toxin activation *in vivo* and, if they do, at which stage of the TA activation cycle such a regulation would occur (Sala et al., 2017; LeRoux et al., 2020). Similarly, it remains to be determined whether or not the control of TA systems by proteolysis relies on a specific activation or induction of proteases (Ramisetty, 2020). Moreover, there is a clear lack of data concerning additional factors such as stress-induced protease adaptors and chaperones, or specific environmental stimuli (or perhaps cell cycle events and other host factors) that might trigger antitoxin degradation and the subsequent toxin activation and/or expression of the TA operon.

Many toxins of *M. tuberculosis* have been cloned, overexpressed and shown to be toxic (Ramage et al., 2009; Sala et al., 2014; Agarwal et al., 2018; Akarsu et al., 2019). Remarkably, several of these toxins were capable of efficiently inducing cell death and their respective antitoxins were essential for *M. tuberculosis* growth (Fivian-Hughes and Davis, 2010; Schuessler et al., 2013; DeJesus et al., 2017; Freire et al., 2019; Cai et al., 2020; Zaveri et al., 2020). This suggests that proteolysis has to be tightly regulated in order to avoid unwanted proteolysis of antitoxins, which could be detrimental for *M. tuberculosis* growth. Yet, under certain conditions, a transient growth inhibition might be beneficial for the pathogen, especially for the entry into a persistent mode.

Finally, the fact that a significant number of toxins were shown to interact with proteases (both ClpC1/P1P2 and the proteasome) suggests that proteolysis could ensure that deleterious toxins do not accumulate. Most of these toxins are part of type 2 TA systems, suggesting that that under certain conditions, inhibition by their cognate antitoxins might not be robust enough without additional control of the toxin by proteolysis. Although new drug discovery strategies that focus on inhibiting mycobacterial proteases seem promising (Lupoli et al., 2018), it is important to note here that such a potentially dual role of proteases on toxin activation or inhibition in *M. tuberculosis* could lead to unwanted toxin activation and the subsequent entry into a persistent mode. Intriguingly, we also noticed that except for DarTG, none of the toxins and antitoxins that interact with proteases are part of the same TA pairs (Figure 1), suggesting a highly complex network of interactions and antagonistic effects that could impact growth of the pathogen in respond to specific signals. More work is needed to uncover such a complex reservoir of interactions involving highly conserved proteolysis pathways and the multiple stress-responsive TA systems of *M. tuberculosis*.
